# Frequent co-expression of EGFR and NeuGcGM3 ganglioside in cancer: it’s potential therapeutic implications

**DOI:** 10.1007/s10585-016-9811-0

**Published:** 2016-07-23

**Authors:** Addys González Palomo, Rancés Blanco Santana, Xiomara Escobar Pérez, Damián Blanco Santana, Mariano Rolando Gabri, Kalet León Monzon, Adriana Carr Pérez

**Affiliations:** 1 Center of Molecular Immunology (CIM), 216 Street and 15 Avenue, Atabey, Playa, P.O. Box 16040, Habana, Cuba; 2 Department of Cell Biology and Tissues Banking, National Institute of Oncology and Radiology, 29 and F Street, Vedado, Plaza de la Revolución, P.O. Box 10400, Havana, Cuba; 3 Laboratory of Molecular Oncology, Quilmes National University, R. Sáenz Peña 180, P.O. Box B1876BXD, Buenos Aires, Argentina

**Keywords:** EGFR, NeuGcGM3, Co-expression, Pulmonary metastasis, Combination therapy

## Abstract

Interaction between epidermal growth factor receptor (EGFR) signaling with GM3 ganglioside expression has been previously described. However, little is known about EGFR and NeuGcGM3 co-expression in cancer patients and their therapeutic implications. In this paper, we evaluate the co-expression of EGFR and NeuGcGM3 ganglioside in tumors from 92 patients and in two spontaneous lung metastasis models of mice (Lewis lung carcinoma (3LL-D122) in C57BL/6 and mammary carcinoma (4T1) in BALB/c). As results, co-expression of EGFR and NeuGcGM3 ganglioside was frequently observed in 63 of 92 patients (68 %), independently of histological subtype. Moreover, EGFR is co-expressed with NeuGcGM3 ganglioside in the metastasis of 3LL-D122 and 4T1 murine models. Such dual expression appears to be therapeutically relevant, since combined therapy with mAbs against these two molecules synergistically increase the survival of mice treated. Overall, our results suggest that NeuGcGM3 and EGFR may coordinately contribute to the tumor cell biology and that therapeutic combinations against these two targets might be a valid strategy to explore.

## Introduction

Most epithelial tumors overexpress the EGFR and their activation is related with cancer progression. However, tumors exhibit a diverse response to anti-EGFR therapies, with resistance as common result of the treatment [1].

The N-Acetylneuramic acid (NeuAcGM3) ganglioside, but not the N-glycolylneuramic acid (NeuGcGM3), is usually detected in normal human tissues. However, many human tumors express NeuGcGM3 ganglioside [2–7]. The expression of NeuGcGM3 have been associated with a worse prognosis in colon [8] and lung cancer [7, 9]. Differential association between EGFR signaling pathway and GM3 ganglioside expression has been reported [10–13]. Overexpression of GM3 increase the proliferation of carcinoma cells (A431) by ERK-independent signaling, in the presence of urokinase plasminogen activator (uPA) and their receptor (uPAr) [14]. Conversely, GM3 depletion increase the EGFR phosphorylation and the ERK-dependent cell proliferation becomes prevalent [14]. These results provide a rational for a combined treatment targeting simultaneously both EGFR and GM3 mediated signaling pathways.

The Center of Molecular Immunology (CIM, Havana, Cuba) have developed several immunotherapeutic projects targeting separately both EGFR [15, 16] and NeuGcGM3 [17, 18]. Therefore, we evaluate the frequency of co-expression of EGFR and NeuGcGM3 ganglioside in human tumors and in two spontaneous lung metastasis models of mice (Lewis lung carcinoma (3LL-D122) in C57BL/6 and mammary carcinoma (4T1) in BALB/c). Moreover, we perform an initial evaluation of the therapeutic implications of targeting simultaneously both molecules, in lung models.

## Materials and methods

### Patients samples

Sections of formalin-fixed and paraffin-embedded tumor tissues from 92 patients were taken from the pathology department of the National Institute of Oncology and Radiobiology and Dr. ¨Manuel Fajardo¨ General Teaching Hospital after receiving approved consent by the Ethical Committee of the institute.

### Cell lines

Lewis lung carcinoma (3LL-D122); mouse breast adenocarcinoma cells (4T1); human vulva epidermoid carcinoma (A431, ATCC, CRL-1555) and murine myeloma P3-X63-Ag8.653 (X63, ATCC, CRL-1580) were cultured in DMEM: F12 (Life Technologies Inc., Grand Islan, NY) supplemented with 10 % fetal bovine serum (FBS).

### Lung metastasis murine models

Mice female of 6–8 week old female, were purchased from the Center for Laboratory Animal Production (CENPALAB, Havana, Cuba). Animal’s procedures were performed in accordance with the guidelines stipulated by Animal Subject Committee Review Board of the CIM and CIM”s Institutional Animal Care and Use Committees. 3LL-D122-metastasis model: C57BL/6 mice were injected into lateral tail veins (i.v.) with 2.5 × 10^5^ of tumor cells. 4T1-metastasis model: BALB/c mice were transplanted subcutaneously (s.c). into the mammary gland with 1 × 10^4^ of tumor cells. Primary tumor diameters were measured every 2–3 days with a caliper and tumor volume (mm^3^) was determined to the following formula = (minor diamenter)^2^ × (major diameter) × π/6. To study overall survival (OS), animals were monitored every day until the primary tumor exceeded 20 % of the body mass (4T1-model) and the signs of malignancy appeared. In parallel experiments, the mice were sacrificed 21 days (3LL-D122-model) and 25 days (4T1-model) after tumor implantation to evaluate lung metastases. Metastatic lung were removed and metastases were quantified through lung weight, established as a surrogate of the number and size of metastasis. Control groups received PBS.

### Murine samples

Tumor sections from the metastatic lungs were obtained by cryostat (SLEE MEDICAL GMBH Co. Mainz, Germany) and mounted on plus slides. Afterwards, in both cases, the slides were kept at −20 °C until they were used for immunostaining.

### Monoclonal antibodies

Ior egf/R3 (R3m) is a mAb against human EGFR [19]. 7A7 mAb for murine EGFR [20]. 14F7 mAb against NeuGcGM3 ganglioside and it was used in patients and murine samples [21]. Regarding the treatment: 14F7 mAb and 7A7 mAb were administered on days 3, 5, 7, 9, 11 and 13 post-inoculation tumoral. 14F7 mAb was used at doses of 200 µg and 7A7 mAb (56 µg), both by i.v.

### Immunohistochemical staining

The slides were incubated with the appropriately diluted primary antibody for 1 h at room temperature. Negative controls were performed by substituting primary for the TBS (Tris/saline buffer solution). Each staining included a known positive control: to human EGFR: A431 cells; to murine EGFR: 3LL-D122 and to NeuGcGM3: X63 cells. Breast ductal carcinoma is positive tissue for human EGFR and NeuGcGM3. R3m, 7A7 and 14F7 mAbs were visualized using the kit Universal Dako LSBA+ System-HRP (Dako, K0679) and the manufacturer’s recommendations were followed. Finally, the sections were counterstained with Mayer’s hematoxylin.

### Evaluation of immunostaining

Results were evaluated by the intensity of immunoreactions and the percentage (%) of the tumor cells displaying immunoreactivity with the cytoplasm and on the membrane pattern. The staining intensity was graded on a scale of 0–3 (0, no staining; 1, weak; 2, moderate and 3, strong staining). The proportion of stained cells was graded on a scale of 0–3 (0, no staining; 1, 6–25 %; 2, 26–50 % and 3, more than 50 %). Two independent observers analyzed five fields in randomly selected tumor areas/animals.

### Double immunofluorescence staining

Co-expression of EGFR and NeuGcGM3 ganglioside was determined by a double staining. R3m or 7A7 mAbs followed by polyclonal rabbit anti-mouse immunoglobulins/FITC (Dako, F0232). 14F7 mAb followed by polyclonal goat anti-mouse immunoglobulins/RPE (Dako, R0480). Co-expression was detected by yellow fluorescence using the Olympus BX51 LTD microscope (Olympus Optical Co., Ltd., Japan).

### Statistical analyses

Overall survival (OS) analyze was performed according to the Kaplan–Meier methods. Statistical analysis was carried out using SPSS (version 11.5, SPSS Inc., Chicago, IL). A p value, 0.05 was considered statistically significant.

## Results

### Co-expression of EGFR and NeuGcGM3 molecules in human primary tumors

**Fig. 1:**
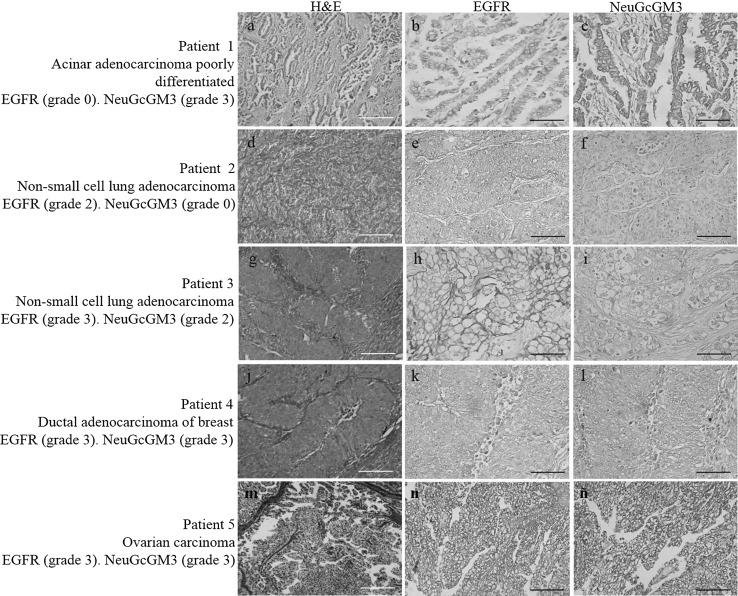
Images of the staining intensity of EGFR and NeuGcGM3 ganglioside in five different patients. These five patients illustrate the most frequent classes observed in our study. Left column shows Hematoxylin and eosin (H&E)-stained tumor tissue sections. Middle and right column show respectively the EGFR and NeuGcGM3 expression (*brown color*). Each row corresponds to the individual patient data specified on the left. EGFR was identified with Ior/egf/r3 mAb and it was mainly located in the cell membrane of malignant cells. NeuGcGM3 ganglioside expression was identified with 14F7 mAb and it was located in cell membrane and cytoplasmatic cell. Counterstaining with Mayer’s Hematoxylin (*blue color*). *White bar* = 50 μm, *Black bar* = 100 μm. (Color figure online)

**Fig. 2:**
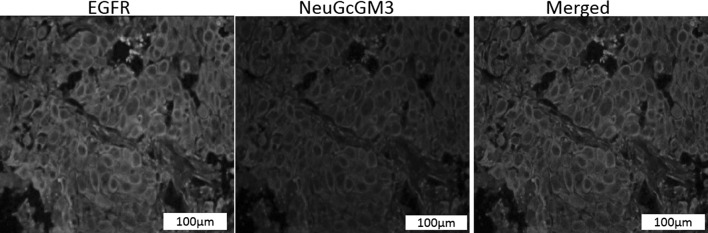
Co-expression of EGFR and NeuGcGM3 ganglioside in human non-small cell lung cancer (poorly differentiated ductal adenocarcinoma). The co-expression of EGFR and NeuGcGM3 was evaluated by a double immunofluorescence staining. Expression of both molecules was located on plasmatic membrane. EGFR-positive section (*green*). NeuGcGM3 ganglioside-positive section (*red*). The *yellow* colour in merged images identifies co-expression of both molecules on tumour sections (merged). *Scale bars* = 100 µm. (Color figure online)

Co-expression of EGFR and NeuGcGM3 ganglioside on tumor samples (63) from 92 patients (68 %) is summarized in Table 1 and exemplified in Fig. 1. First, EGFR and NeuGcGM3 expression was analyzed by immunohistochemistry and second, the co-expression of both molecules was determined by immunofluorescence assay. The intensity of immunoreactions and proportion of stained cells was variable. A weak to strong intensity staining following a homogeneous cell membrane pattern in 68 of the 92 (74 %) of EGFR was detected. Similar intensity of NeuGcGM3 ganglioside expression with plasmatic membrane and cytoplasm staining was observed in 71 (77 %). The tumors with the greatest number of patients (n > 6): NSCLC, nasopharyngeal, colorectal, renal and liver carcinoma showed co-expression (EGFR/NeuGcGM3) in more than 50 % of their tumoral cells. Other carcinomas with fewer patients (n = 2–5) also showed high percentage of EGFR/NeuGcGM3 positive cells: prostate, ovarian, cervical, breast carcinoma and glioblastoma multiform. In addition, co-expression of both EGFR and NeuGcGM3 ganglioside (30-70 %) was observed in others tumors with small number of patients but of different origin as sarcomas (n = 3) and non-Hodgkin lymphomas (n = 3). Representative image of co-expression of EGFR and NeuGcGM3 molecules is observed in Fig. 2.

**Table 1:** Co-expression of EGFR and NeuGcGM3 ganglioside in human primary tumors

Histological subtype	EGFR						NeuGcGM3						Double positive
	Staining intensity	Positive cells					Staining intensity	Positive cells					
		0	1	2	3	Total (%)		0	1	2	3	Total (%)	Total (%)
NSCLC						13/23 (56)						15/23 (65)	12/23 (52)
Adenocarcinoma (ADC)	2–3	4	2	2	3	7/11 (64)	1–3	4	1	3	3	7/11 (64)	6/11 (54)
Squamous cell carcinoma	2	6	1	2	3	6/12 (50)	1–3	4	1	4	3	8/12 (67)	6/12 (50)
Digestive system						30/35 (86)						31/35 (88)	31/35 (88)
Stomach (ADC)	2–3	0	0	2	1	3/3 (100)	2–3	1	0	1	1	2/3 (67)	2/3 (67)
Nasopharyngeal carcinoma	1–3	4	3	4	3	10/14 (71)	1–3	1	2	3	8	13/14 (93)	10/14 (71)
Colorectal (ADC)	1–3	0	0	2	6	8/8 (100)	1–3	1	0	4	3	7/8 (87)	7/8 (87)
Pancreas (ADC)	2–3	0	0	0	1	1/2 (50)	2–3	0	0	0	2	2/2 (100)	1/2 (50)
Liver	2–3	0	0	1	7	8/8 (100)	2–3	1	0	4	3	7/8 (87)	7/8 (87)
Urogenital system						16/23 (69)						19/23 (83)	14/23 (61)
Prostate	1–3	2	0	1	2	3/5 (60)	1–3	1	0	1	3	4/5 (80)	3/5 (60)
Ovary	2	1	1	1	1	4/5 (80)	3	3	1	1	0	2/5 (40)	2/5 (40)
Cervical carcinoma	3	0	0	0	2	2/2 (100)	3	0	0	0	2	2/2 (100)	2/2 (100)
Breast carcinoma	3	3	0	0	2	2/5 (40)	3	0	0	3	2	5/5 (100)	2/5 (40)
Renal cell carcinoma	1–3	1	0	3	2	5/6 (83)	3	0	3	2	1	6/6 (100)	5/6 (83)
Nervous system						5/5 (100)						3/5 (60)	3/5 (60)
Glioblastoma multiforme	3	0	0	3	2	5/5 (100)	2	0	2	1	0	3/5 (60)	3/5 (60)
						2/3 (67)						1/3 (33)	1/3 (33)
Sarcomas	1–2	1	2	0	0	2/3 (67)	1	2	1	0	0	1/3 (33)	1/3 (33)
Haemopoietic system						2/3 (67)						2/3 (67)	2/3 (67)
Non-hodking lymphoma	1	1	2	0	0	2/3 (67)	1	1	2	0	0	2/3 (67)	2/3 (67)

Staining intensity: 0, no staining; 1, weak; 2, moderate and 3, strong staining

Positive cells: 0, no staining; 1, 6–25 %; 2, 26–50 % and 3, more than 50 %

### Co-expression of EGFR with NeuGcGM3 molecules in murine models

**Fig. 3:**
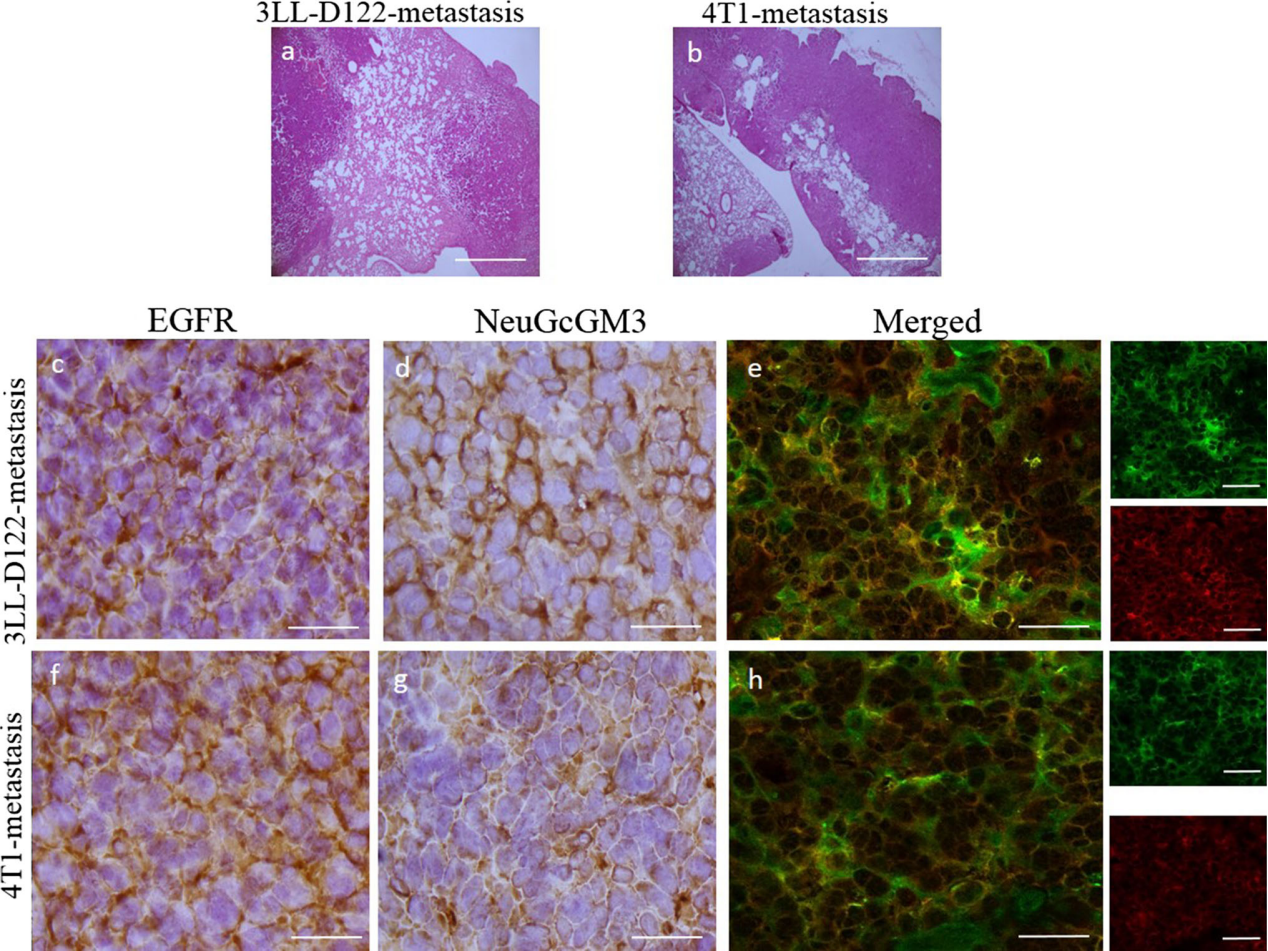
Co-expression of EGFR with NeuGcGM3 molecules on lung metastasis. C57BL/6 mice bearing the Lewis Lung carcinoma (3LL-D122-model) or BALB/c mice bearing the breast carcinoma (4T1-model). Histologic section of representative lung metastasis originating from 3LL (*a*) and 4T1-model (*b*) by hematoxilin and eosin staining (H/E). Metastatic tumors throughout the lung parenchyma in both lung models. EGFR *(c*) and NeuGcGM3 (*d*) expression on 3LL-D122 or 4T1 (*f* and *h*) tumor sections were characterized by immunostaining assay. Co-expression of both molecules on lung sections was evaluated by a double immunofluorescence method: 3LL-model (*e*) and 4T1-model (*h*). EGFR-positive section (*green*). NeuGcGM3 ganglioside-positive section (*red*). The yellow color identifies co-expression of both molecules on tumor cells (merged) in 3LL-model (e) and 4T1-model (*h*). *White bars* = 100 µm. (Color figure online)

First, EGFR and NeuGcGM3 ganglioside expression on tumor sections from Lewis Lung carcinoma (3LL-D122) and breast carcinoma (4T1) were characterized by immunohistochemistry assay: Table 2 and Fig. 3. EGFR and NeuGcGM3 ganglioside expression was detectable in 30 of 30 (100 %) animals in both models (Table 2). EGFR and NeuGcGM3-immunostaining was located in the plasmatic membrane and/or cytoplasm staining finely granular pattern (Fig. 2: 3LL-model (a and b) and 4T1-model (d and e). Table 2 shows a moderate to strong staining of EGFR and weak to strong intensity to NeuGcGM3 in 3LL-D122 model. Most specimens had strong intensity to EGFR (17 of 30 animals, 57 %) and to NeuGcGM3 (14, 47 %). 4T1-model showed a weak to strong reactivity of EGFR and NeuGcGM3 (Table 2). The intensity of EGFR expression in 13 (44 %) of 30 animals is strong while the most animals 14 (47 %) shown a weak reactivity to NeuGcGM3 ganglioside. Double-expression of EGFR (e) and NeuGcGM3 (h) was detected in both models, Fig. 3.

**Table 2:** Co-expression of EGFR and NeuGcGM3 ganglioside in murine models

	EGFR						NeuGcGM3						Double positive
	Positive cells						Positive cells						
Metastasis model	Staining intensity	0	1	2	3	Total (%)	Staining intensity	0	1	2	3	Total (%)	Total (%)
3LL-D122-cells	2–3	0	0	1	29	30/30 (100)	1–3	0	1	3	26	30/30 (100)	30/30 (100)
4T1-cells	1–3	0	0	2	28	30/30 (100)	1–3	0	2	9	19	30/30 (100)	30/30 (100)

Staining intensity: 0, no staining; 1, weak; 2, moderate and 3, strong staining

Positive cells: 0, no staining; 1, 6-25 %; 2, 26-50 % and 3, more than 50 %

### Therapeutic implications of EGFR and NeuGcGM3 expression in murine models

**Fig. 4:**
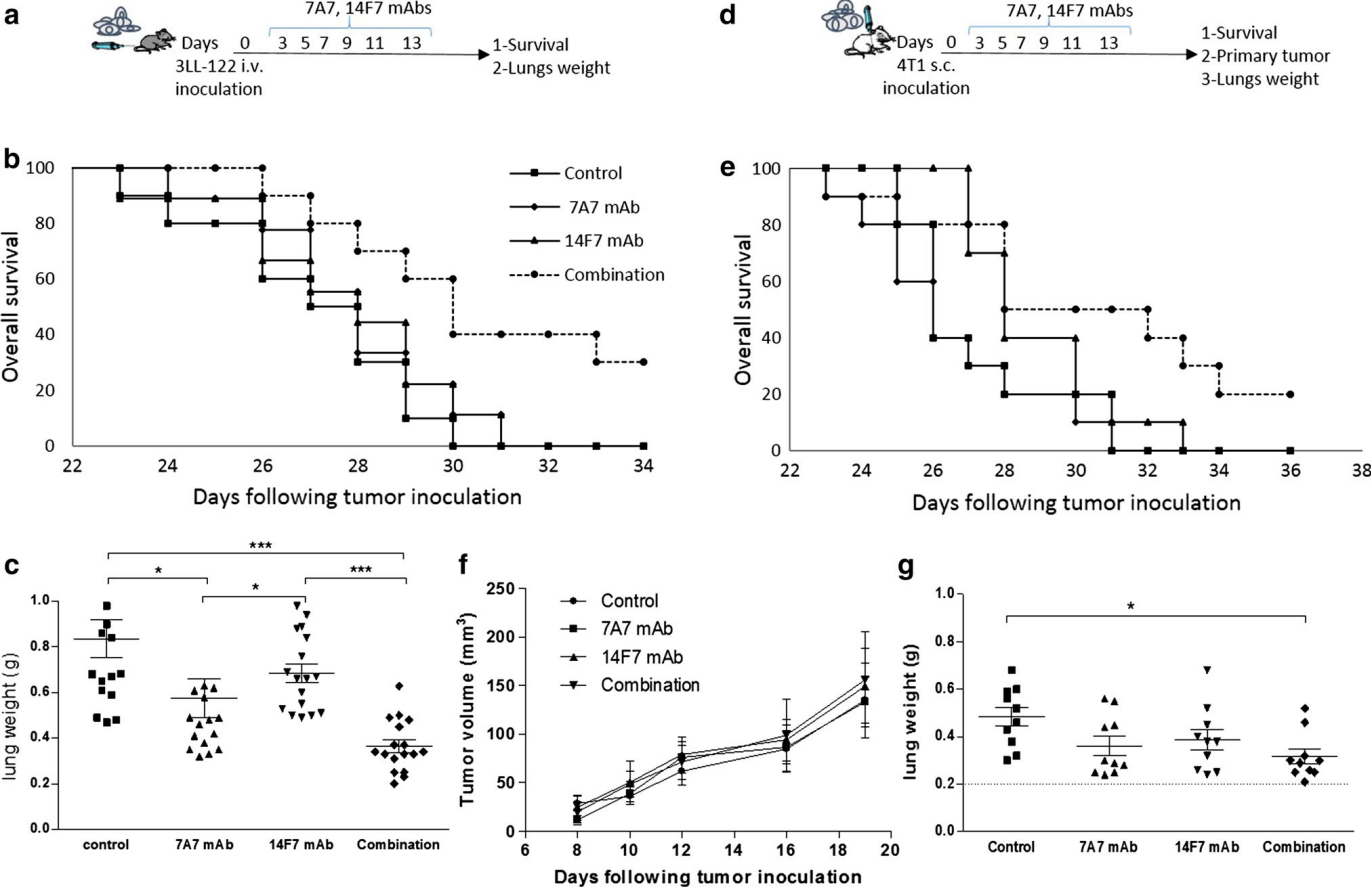
Combinatorial targeted therapy to the EGFR and NeuGcGM3 ganglioside in two lung metastasis models of mice: Lewis lung carcinoma (3LL-D122) in C57BL/6 (*a-c*) and mammary carcinoma (4T1) in BALB/c (*d-g*) . Mice were challenged i.v. (2.5 × 10^5^) with 3LL or subcutaneously (1 × 10^4^) with 4T1 cells. Administration of schedule with 7A7 or 14F7 mAbs (i.v.) is indicated in schematic representation of metastasis assays (*a* and *d*). To analyze the percentage of survival, animals (n = 10 per group) of both models were monitored every day. Kaplan–Meier curves of overall are showed for each metastasis assay, (p < 0.05, Log-rank test). Pulmonary metastases were measured as lung weight on both lung metastasis models: 3LL-model (*c*) and 4T1-model (*g*). Normal lung weight value is indicated (*dashes lines*). Primary tumor volume in 4T1-model (*f*). Each point represents mean ± SD of lung weight per animals in each group of mice (*c* and *f*). One representative experiment out of three performed experiments is shown in each case

To explore the therapeutic impact of targeting simultaneously EGFR and NeuGcGM3 molecules in tumors, we treat 3LL (Fig. 4a–c) or 4T1 (Fig. 4d–g) metastasis-bearing mice with a combination of 7A7 mAb with 14F7 mAb. The survival behavior by monotherapies effect was similar to control group (Fig. 4b, e). However, a significant increase was observed by combined therapy (log rank p < 0.05) (Fig. 4b, e). 3LL-model showed a 30 % of the mice remain alive (Fig. 4b). 4T1-model showed a similar result (25 %) (Fig. 4e). In parallel experiments, the effect of combined therapy on lungs weight and/or primary tumor was evaluated at day 21 (3LL) or day 25 (4T1) post-tumor inoculation. In 3LL-model, 7A7 mAb and combined therapy reduce the lungs weight compared with 14F7 mAb and control group Fig. 4c. However, not difference was observed between them. Regarding 4T1-model, neither individual nor combined treatment had impact on primary tumor (Fig. 4f). In addition, only combined therapy showed a reduction of the lungs weight compare with control group (Fig. 4g).

## Discussion

In this paper, we report for the first time the co-expression of EGFR and NeuGcGM3 ganglioside in different human tumors. In fact, more than 50 % of co-expression of EGFR and NeuGcGM3 from 15 histological subtypes was observed. Our results suggest that it is a frequent phenomenon and thus may support diagnostic considerations. Recently, our colleagues at CIM confirm that the expression of NeuGcGM3 or EGFR alone was associated with poor OS in NSCLC patients [9]. However, the expression of both targets in the same tumor sample was related with an even poorer prognosis in these patients [9]. On the other hand, the heterogeneity of EGFR and NeuGcGM3 ganglioside expression (over a range of negative to strong immunostaining and more than 50 % of positive cells) was consistent with others reports by each molecules, independently: EGFR [22, 23] or NeuGcGM3 [2, 7, 8]. In addition, this differential expression pattern implies heterogeneity within individual tumor and may be reflected in the biological behavior of these tumors.

To explore a possible relationship between EGFR and NeuGcGM3 in cancer, we first evaluate their expression in two models of spontaneous lung metastasis in mice: Lewis lung carcinoma (3LL-D122) and mammary carcinoma (4T1). We confirm a high EGFR expression in 3LL-D122 and 4T1-metastatic models by immunohistochemistry score. Regarding to NeuGcGM3 expression, all animals showed a positive tumor cells, in both models. Previously, Labrada et al. showed the increased expression of NeuGcGM3 from primary tumors to metastatic lesions in 3LL-D122 tumor model [24]. Anti-metastatic effect of NeuGcGM3/VSSP vaccine which targeting this ganglioside was observed in this lung model, previously [24, 25]. Here, we described for the first time, a positive staining of NeuGcGM3 expression in lung metastasis induced by 4T1-cells, as second model. Similarly, to 3LL-D122, 4T1-cells are negative to NeuGcGM3 ganglioside expression, in vitro (data not shown). Moreover, the intensity of NeuGcGM3 expression was slightly lower than metastasis from 3LL-model. Future studies going on carrying out to characterize the expression of NeuGcGM3 in others organs colonized by 4T1-cells different methods. Interestingly, EGFR and NeuGcGM3 ganglioside are co-expressed at the plasmatic membrane in both models. These murine scenarios are a useful tool for exploring therapeutic combinations.

Second step, the combination of 7A7 mAb (anti-EGFR) and 14F7 mAb (anti-NeuGcGM3) exhibited a synergistic therapeutic effect on the OS in 3LL-D122 or 4T1 lung metastasis-bearing mice. Anti-tumor effect of the individual therapies with 7A7 mAb [20, 26, 27] and 14F7 mAb [21, 28] has been previously reported. Survival benefits of 14F7 mAb were only observed in tumor models from hematological malignancies. The combination therapy effect in OS of 4T1-model was smaller compared with the results in 3LL-model, that fact could be connected with the persistence of the primary tumor or heterogeneous expression of NeuGcGM3 in lung metastases.

The approach was the combination of the tumor-cell oncosis and antibody dependent cell-mediated cytotoxicity by 14F7 mAb [28, 29] with the inhibition of proliferation, pro-apoptosis effect and the involvement of T cells in the antitumor activity of 7A7 mAb [20, 26, 27]. The survival data could be support by complementary mechanisms mentioned before. Our data are agree with others combined therapies based in monoclonal antibodies [30, 31]. These results could be translated to clinical scenery as the opportunity to combine anti-NeuGcGM3 therapy with Nimotuzumab (Ior egf/r3, anti-EGFR mAb) that have been demonstrating safety during long period of treatments [32, 33].

In summary, our results suggest that co-expression of EGFR and NeuGcGM3 is frequent in human tumors. These two molecules might coordinately contribute to the tumor cell biology and therefore the combinations of therapies against these two targets might be a valid strategy to explore in the clinic. The impact of EGFR and NeuGcGM3 co-expression as a prognosis factor in human tumors will are exploring, and the mechanisms mediating the synergistic effect of the combination therapy shall be further evaluated.
